# Simultaneous introgression of three *POLLED* mutations into a synthetic breed of Chinese cattle

**DOI:** 10.1371/journal.pone.0186862

**Published:** 2017-10-20

**Authors:** Shi-Yi Chen, Linhai Liu, Maozhong Fu, Gong-Wei Zhang, Jun Yi, Song-Jia Lai, Wei Wang

**Affiliations:** 1 Farm Animal Genetic Resources Exploration and Innovation Key Laboratory of Sichuan Province, Sichuan Agricultural University, Chengdu, China; 2 Sichuan Animal Science Academy, Chengdu, China; 3 College of Animal Science, Southwest University, Chongqing, China; National Cheng Kung University, TAIWAN

## Abstract

The polled phenotype of cattle is increasingly becoming favourable mainly because of the enhanced emphasis on animal welfare, for which the causative mutations have been reported during the past years. The Shuxuan cattle are a new synthetic breed by crossing the indigenous cattle with both Simmental and Holstein semen in Sichuan of Southwest China, in which about 15% of polled individuals have newly emerged. Because official record about *POLLED* genotypes for the historically imported sires is unavailable, we therefore genotyped the proposed *POLLED* variants of P_202ID_, P_80kbID_ and P_219ID_ among 48 polled and 16 horned Shuxuan cattle. It was first revealed that all three candidate mutations have been simultaneously introgressed into Shuxuan cattle, whereas the P_202ID_ mutation is dominant. Furthermore, one polled animal still remains to carry none of the three candidate mutations, which suggests that further mutation(s) would also exist. Additionally, we sequenced mitochondrial DNA and found that Shuxuan cattle are composed of two matrilineal origins of *Bos taurus* (65.6%) and *B*. *indicus* (34.4%); and there is no origin-biased distribution of polled phenotype. In conclusion, our study first supports the recently reported novel candidate mutation of P_219ID_ and detects simultaneous presences of all three known *POLLED* mutations within a cattle breed.

## Introduction

Horns are a distinctive characteristic of animals in *Bovidae* family and exhibit very high morphological diversity [1]. As a powerful weapon in fight against predators and amongst each other, the individuals that bear strong horns would have evolutionary advantage in the wild [2]. However, breeding hornless cattle is increasingly becoming a favourable alternative in modern husbandry systems due to the improved convenience in herd management practice. Although the genetically hornless individuals (referred to as polled phenotype) have existed for a long time, it still remains rather common to produce hornless cattle by physical dehorning at young age [3]. Because animal welfare in relation to the physical dehorning is an increasing issue of public concern, it is anticipated that hornless cattle will be preferably produced by genetic selection of polled individuals [4].

The polled phenotype of cattle is inherited as an autosomal dominant trait, for which the *POLLED* locus was genetically mapped to Chromosome 1 [5–8]. During the past years, many efforts have been devoted to explore causative mutation(s) for polled phenotype in cattle [9–14] as well as the related bovine species of yak [15, 16]. Two heterogeneous mutations were first observed to perfectly segregate with polled phenotype, including the complex duplication-insertion of 202 bp fragment (P_202ID_) in beef or dual-purpose breeds and 80 kb fragment (P_80kbID_) in Holstein [10, 17]. More recently, a novel duplication-insertion event of a 219 bp fragment (P_219ID_) was also revealed to be responsible for polled phenotype in Mongolian Turano cattle [18]. Therefore, a total of three causative mutations have been confidently proposed to be associated with polled phenotype in cattle so far.

Although none of the three *POLLED* mutations is physically located into any known coding region or contains functional element, it is empirically verified recently by the genome editing technology that P_202ID_ is a causative mutation for ontogenesis of polled phenotype in Holstein [19]. Within the mapped *POLLED* locus, it was first reported that there is no differentially expressed gene between the polled and horned phenotypes of cattle as being revealed by the cDNA microarray technology [20]. Subsequently, a long intergenic non-coding RNA was revealed to be ectopic expression in horn buds of polled cattle with the aid of high-throughput mRNA sequencing technology, which would be involved into the molecular regulation of horn agenesis [12]. However, direct link between these causative mutations and molecular genetic mechanism underlying polledness ontogenesis in cattle still remains to be investigated.

The Shuxuan cattle are a synthetic dual-purpose breed and mainly distributed in Sichuan Province of Southwest China by crossing the indigenous breed of Xuanhan cattle with both Simmental and Holstein semen during the past 30 years. Finally, it is estimated that the cultivated Shuxuan cattle consist of about 75% Simmental and 10% Holstein blood. In contrast to the indigenous Xuanhan cattle that are a completely horned breed, we currently observed that about 15% of Shuxuan cattle are the polled phenotype according to our field investigation. Unfortunately, no official record is available for describing the horn trait or *POLLED* genotype for these historically introduced sires of Simmental and Holstein. Because of allelic heterogeneity of polled phenotype as stated above, we directly genotype the proposed candidate variants for polled Shuxuan cattle in the present study and intend to reveal that which mutation(s) had been introgressed into this newly cultivated breed. The results are essential to establish the marker-assisted selection program of hornless Shuxuan cattle.

## Materials and methods

### Ethics statement

No ethical approval was required in the present study because all blood samples were collected by local veterinarians for annual health inspection.

### Animals and DNA extraction

A total of 64 Shuxuan cattle were collected in the present study, including 48 polled and 16 horned individuals (S1 Table). The polled phenotype for each cattle was accurately approved via both visual and touch inspections to avoid the scurs phenotype. The possible practice of artificial dehorning was also excluded by carefully consulting cattle breeder. However, genetic relatedness remains unknown among all animals. The genomic DNA was extracted using AxyPrep Genomic DNA Miniprep Kit (Axygen Bioscience, USA).

### Genotyping of candidate mutations for polled phenotype

Variant P_202ID_ was first genotyped using the published PCR primers and method [10], which distinguishes different genotypes through agarose gel electrophoresis of PCR products. To avoid potential false-negative PCR outcome, a primer pair was newly designed in the present study to independently amplify an overlapping fragment encompassing variant P_202ID_; and for which PCR products were similarly subjected to the agarose gel electrophoresis-based genotyping. Primer sequences and genotyping methods involved in the present study are detailed in S2 Table.

Subsequently, we genotyped variant P_80kbID_ according to the initially reported method by Medugorac and colleagues [10], in which the surrogate variant P_T1909396D2_ (a two-base pair deletion) for P_80kbID_ was used and genotyped by PCR amplification and capillary electrophoresis. Because three genotypes of variant P_80kbID_ must be discriminated according to both qualitative and quantitative comparisons on the obtained signal, it would be apt to lead to false conclusion of genotyping. Therefore we further subjected this PCR amplified fragment to Sanger sequencing in parallel, by which animals in either homozygous or heterozygous states for mutant allele could be obviously distinguished from wild-type homozygote according to the presence or not of heterogeneous peaks in the chromatographic traces.

To further verify genotyping results of P_80kbID_, four additionally linked variants, including the P_5ID_, P_G1855898A_, P_C1768587A_ and P_G1654405A_, were also genotyped using the Sanger sequencing approach because all of them had been proposed to segregate with P_80kbID_ within a 260 kb haplotype block [10]. Subsequently, we also investigated the linkage disequilibrium by web tool of SHEsis [21] for these five genotyped variants (P_80kbID_, P_5ID_, P_G1855898A_, P_C1768587A_ and P_G1654405A_). According to the recent publication [18], we further genotyped variant P_219ID_ among these polled individuals in which neither P_202ID_ nor P_80kbID_ mutation was observed.

### Mitochondrial DNA sequencing and analysis

To determine genetic composition of Shuxuan cattle, mitochondrial D-loop sequence was amplified and sequenced for all animals (S2 Table). Briefly, the PCR products were purified and partially sequenced using the forward primer, which generated sequences of ~820 bp in length. After the raw sequences were edited and truncated, we aligned them using DNAstar tool (DNAS Inc, Madison, WI, USA). Subsequently, a rooted neighbor-joining phylogenetic tree was constructed using yak (*B*. *grunniens*) as outgroup to discern matrilineal origins of *B*. *taurus* and *B*. *indicus*.

## Results and discussion

**Fig 1:**
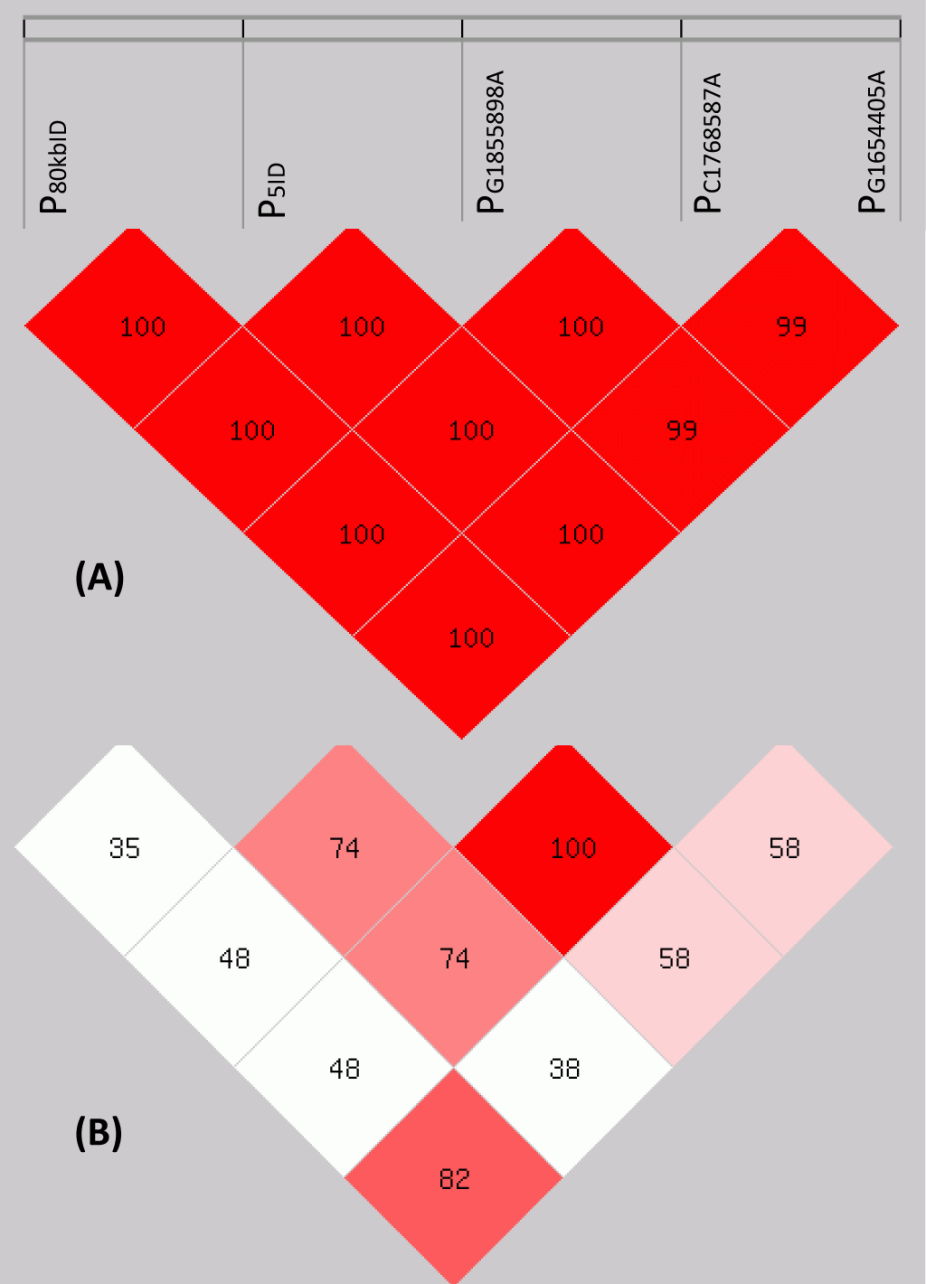
Linkage disequilibrium plots of the five variants of P_80kbID_. Number in each cell is the pairwise Lewontin’s D’ (A) and r^2^ (B), respectively.

We successfully genotyped candidate variants of P_202ID_ and P_80kbID_ for all 64 Shuxuan cattle in the present study (S1 Table). To avoid genotyping error [17], we therefore employed independent genotyping approaches for both two variants. For variant P_80kbID_, we additionally investigated genotypes for the four tightly linked variants, by which the five related variants were finally revealed to be in almost complete linkage disequilibrium in Shuxuan cattle (Fig 1). This result is consistent with previous report [17] and therefore supports our genotyping reliability of P_80kbID_.

All horned Shuxuan cattle are the wild-type homozygotes for both variants of P_202ID_ and P_80kbID_, which are not beyond the observations reported in previous studies [10, 12]. Both P_202ID_ and P_80kbID_ mutations were simultaneously detected among the polled individuals, for which the observed frequencies of mutant alleles are 43.75% and 8.33%, respectively (Table 1). Therefore, it is concluded that the polled phenotypes in Shuxuan cattle are genetically determined by both P_202ID_ and P_80kbID_ mutations, which, however, is predominated by P_202ID_. The results are consistent with the fact that Shuxuan cattle currently consist of about 75% Simmental and 10% Holstein blood. Furthermore, there is one animal in which both P_202ID_ and P_80kbID_ mutations are recombined together, which was similarly reported by Rothammer and colleagues [17].

**Table 1:** Genotypes and alleles for P_202ID_ and P_80kbID_ among polled individuals.

Variants	Genotypes			Alleles	
	W/W	W/M	M/M	W (%)	M (%)
**P**_**202ID**_	9	36	3	56.25	43.75
**P**_**80kbID**_	44	0	4	91.67	8.33

W, wild-type allele; M, mutant allele.

**Fig 2:**
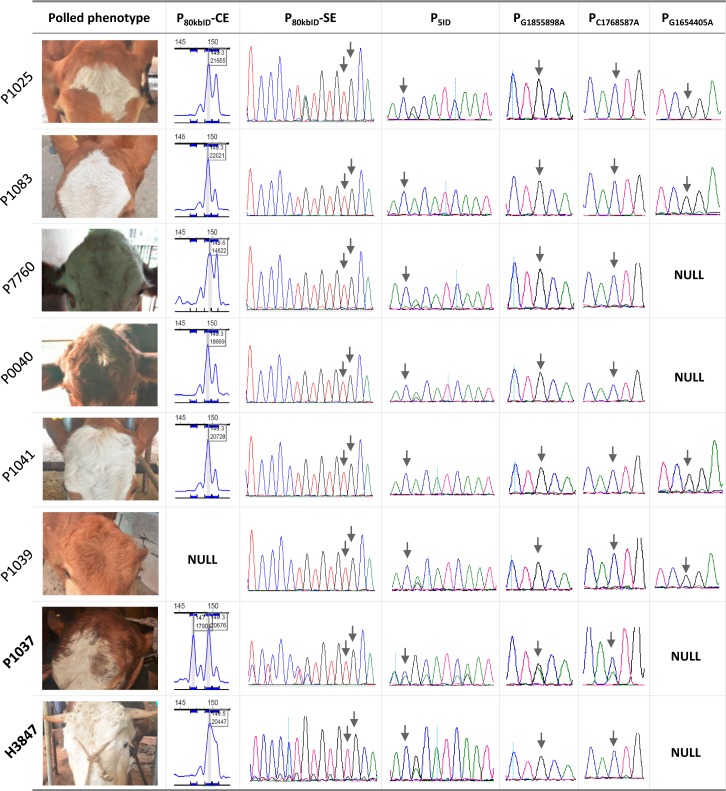
Genotyping of P_80kbID_ and the linked variants. Photos show the polled phenotypes for the six cattle that bear wild-type homozygotes for both P_202ID_ and P_80kbID_. One polled cattle (P1037) in homozygous state of mutant allele of P_80kbID_ and one horned animal (H3847) are illustratively provided in the last two rows. By the capillary electrophoresis method (P_80kbID_-CE), a single peak at 149 bp would be visualized for the wild-type homozygote of P_80kbID_; otherwise it will result into two peaks at 147 bp and 149 bp, respectively. Sanger sequencing of the fragments encompassing P_80kbID_ (P_80kbID_-SE) and P_5ID_ variants would produce heterozygous chromatographs due to presence of InDel mutation. The initially reported mutations are denoted by arrows. NULL also indicate failure in genotyping.

However, there still remain six polled Shuxuan cattle that carry neither P_202ID_ mutation nor P_80kbID_ mutation (S1 Table and Fig 2). Although tens of cattle breeds and thousands of individuals have been comprehensively genotyped for this two candidate variants in previous studies, only one Holstein sire in polled phenotype was finally revealed to carry neither of them [10, 11, 17]. Accordingly, we initially speculated that there would still be unknown mutation(s) associated with the polled phenotype in Shuxuan cattle. During the preparation of this manuscript, however, we found that Medugorac and colleagues [18] much recently reported the third candidate mutation (P_219ID_) of polled phenotype independently observed in Mongolian Turano cattle. Therefore, the six polled Shuxuan cattle were again subjected to genotyping of the novel P_219ID_, by which we newly found that five cattle are heterozygous genotypes for this mutation. However, one animal (P0040) still remains to be wild-type homozygote for the variant P_219ID_. The Mongolian Turano cattle are mainly distributed in North Eastern Asia and suggested to contain a specific mitochondrial DNA haplogroup of T4 that would have been locally domesticated [22]. We subsequently classified the two polled Shuxuan cattle of *B*. *taurus* which also carry P_219ID_ mutation and revealed that they belong to T2 rather than T4 haplogroup. Additionally, there is no official record for the possible paternal introgression of gene pool of Mongolian Turano cattle into Shuxuan cattle. Therefore, the presence of P_219ID_ mutation in Shuxuan cattle can’t be explained by the acknowledged breeding process. An alternative possibility is that P_219ID_ mutation would be also present in other breeds, which had ever been imported into the Shuxuan cattle.

Although all three proposed *POLLED* mutations were simultaneously detected in Shuxuan cattle, there still remains a polled animal that doesn’t carry any one of them yet. This observation raises our presumption that unknown causative mutation(s) of polled phenotype in cattle would also exist. In fact, it would have become conclusive that both P_202ID_ and P_80kbID_ are perfectly associated with polled phenotype after both of them had been comprehensively genotyped in a large panel of European cattle breeds [17]. However, it becomes again an open question due to novel finding of the third candidate mutation of P_219ID_ in Mongolian Turano cattle [18]. More importantly, all the three variants of P_202ID_, P_80kbID_ and P_219ID_ are referred to as the mutation type of fragment duplication-insertion and also adjacently located within a ~270 kb block. It would therefore be reasonable to deduce that similar mutation within or around this block region also would likely cause the polled phenotype in cattle, which should be investigated in the future.

In addition to the genetic variants, a phenotypic character of atypical eyelashes was also proposed to be perfectly associated with the polled phenotype in cattle [12]. Unfortunately, we did not examine this visual phenotype for all individuals sampled in the present study. Another critical question is the molecular mechanism underlying phenotypic ontogenesis of polledness in cattle, to which a few studies have been specially paid via investigating the differentially expressed genes between polled phenotype and wild-type [12, 13, 20]. However, only limited evidence has been effectively elucidated possibly because of low detection power of microarray-based gene expression profiling platform [20] or insufficient biological samples subjected to high-throughput mRNA sequencing [12]. Additionally, it would be very difficult to determine appropriate sampling time points due to the complexity of prenatal horn bud dynamic development [13, 23].

**Fig 3:**
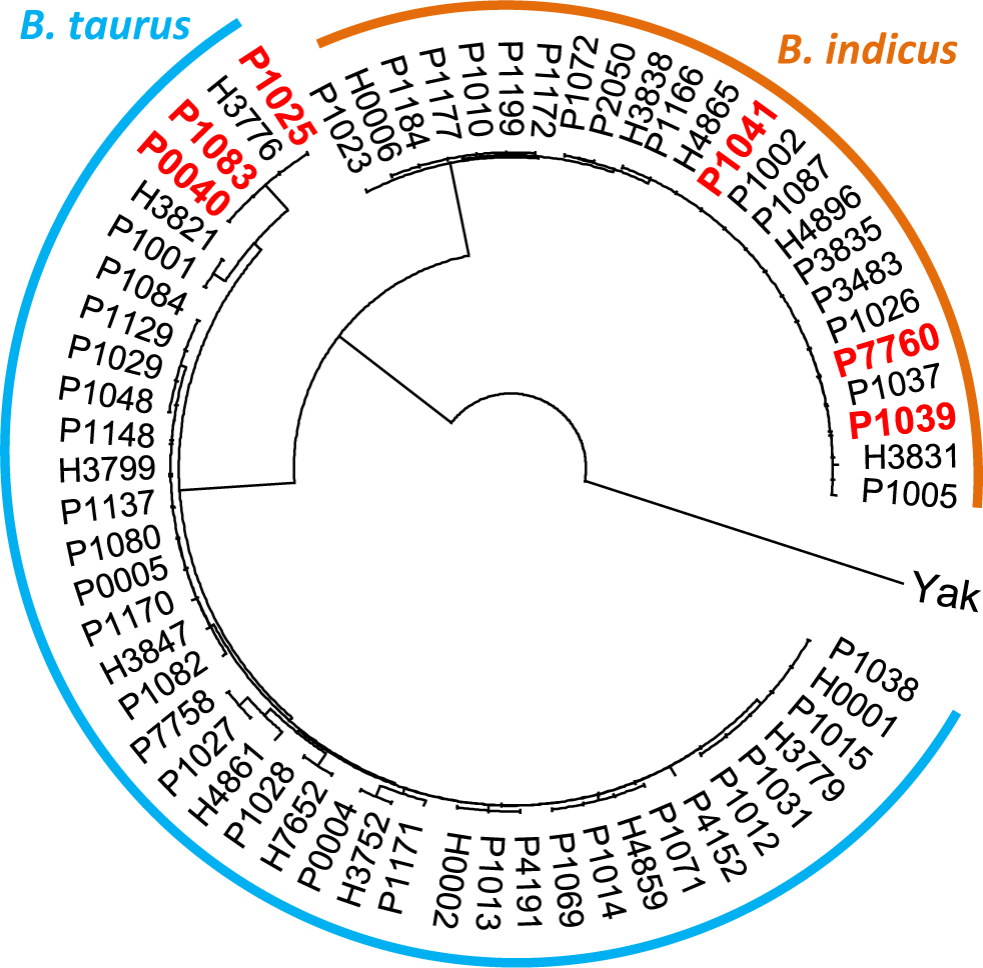
Neighbor-joining tree of mitochondrial D-loop sequences. Two clades are detected with corresponding to *B*. *taurus* and *B*. *indicus*, respectively. Six polled cattle bearing the wild-type homozygotes for both variants of P_202ID_ and P_80bID_ are denoted in red.

The Chinese cattle are evolutionarily composed of two matrilineal origins of *B*. *taurus* and *B*. *indicus* [24]. The indigenous cattle breeds that are distributed in central China, such as Sichuan and Shaanxi provinces, would be essentially mixed by this two components [25]. Therefore, we intended to know whether the presence of polled phenotype in Shuxuan cattle is unevenly distributed between *B*. *taurus* and *B*. *indicus*. By sequencing mitochondrial DNA, a total of 42 and 22 individuals were classified into the *B*. *taurus* and *B*. *indicus*, respectively (Fig 3). Furthermore, all 48 polled Shuxuan cattle consist of 31 *B*. *taurus* and 17 *B*. *indicus* individuals, which herein suggest that there is no matrilineal origin-biased distribution of polled phenotype.

Based on the estimated proportion of 15% of polled individuals in Shuxuan cattle, it can be computed that the total frequency of polled alleles is 8.6% for all the three candidate variants. Furthermore, about 75% of the mutant alleles of polled phenotype are currently carried in heterozygous state. In order to breed a stable polled population in Shuxuan cattle, therefore, the direct selection based on phenotype or even on the genotype would take a long time or result into considerable genetic loss because the polledness trait is inherited in the dominant manner. Fortunately, a recent study first proposed that approach of genomic selection could speed up the selection progress of polled population [26], which could be applied to Shuxuan cattle in the future.

### Conclusion

In the present study, we first reported that all three proposed mutations of polled phenotype of cattle are simultaneously introgressed into a synthetic cattle breed in China. However, one polled animal was revealed to carry none of these candidate mutations, which therefore suggests that further causative mutation(s) of the polled phenotype in cattle would also exist.

## Supporting information

S1 Table Sample information and genotyping results of P_80kbID_ and the related variants. (XLSX)

S2 Table Primers, PCR amplification and genotyping methods used in the present study. (DOCX)
