# Supplementary material for: Simultaneous introgression of three POLLED mutations into a synthetic breed of Chinese cattle
Source: PLoS One. 2017 Oct 20;12(10):e0186862. doi: 10.1371/journal.pone.0186862 (PMC5650179; doi:10.1371/journal.pone.0186862)
Supplement: S2 Table — (DOCX) [file pone.0186862.s002.docx]

**S2 Table** Primers, PCR amplification and genotyping methods used in the present study

| **Candidate mutations** | **Primer sequences (5' - 3’)** | **Binding sites of primer** | **Sources** | **Annealing temperatures** | **Genotyping method** |
| --- | --- | --- | --- | --- | --- |
| **P_202ID_** | F: TCAAGAAGGCGGCACTATCT  R: TGATAAACTGACCCTCTGCCTATA | chr1: 1,705,792 – 1,705,811  chr1: 1,706,160 – 1,706,137 | Ref. [10] | 58 °C | Agarose gel electrophoresis |
|  | F: CGAGGAATGCTTAGAAGTGT  R: ACCCAGAATGTATCGTGAGT | chr1: 1,705,738 – 1,705,757  chr1: 1,706,252 – 1,706,233 | This study | 56 °C | Agarose gel electrophoresis |
| **P_80kbID_** | F: GAAGTCGGTGGTCTGAAAGG  R: TGTTCTGTGTGGGTTTGAGG | chr1: 1,909,358 – 1,909,377  chr1: 1,909,509 – 1,909,490 | Ref. [10] | 59 °C | Capillary electrophoresis |
|  |  |  |  |  | PCR sequencing |
| **P_5ID_** | F: CATCGGCTCTTCATACAATACAATC  R: ACGCTATAGTCAGAGATCACAAAGA | chr1: 1,648,889 – 1,648,913  chr1: 1,649,500 – 1,649,476 | This study | 59 °C | PCR sequencing |
| **P_G1855898A_** | F: TCCAGCAATGAGACAAAATG  R: TCCATCTAATCTCCCCCTGTT | chr1: 1,855,617 – 1,855,636  chr1: 1,856,116 – 1,856,096 | Ref. [12] | 55 °C | PCR sequencing |
| **P_C1768587A_** | F: AGGAGGTTGGCATTTGATTG  R: AAATCCAGAGTTGAGCCGAT | chr1: 1,768,264 – 1,768,283  chr1: 1,768,855 – 1,768,836 | This study | 58 °C | PCR sequencing |
| **P_G1654405A_** | F: GCAATAACAAAACCGAAAGCAGGAT  R: CTGAACTTACAGGTGAAGGAATCTG | chr1: 1,654,055 – 1,654,079  chr1: 1,654,697 – 1,654,673 | This study | 60 °C | PCR sequencing |
| **D-loop** | F: CTGCAGTCTCACCATCAACC  R: GATTATAGAACAGGCTCCTC | MT: 15,768 – 15,787  MT: 592 – 573 | Ref. [25] | 58 °C | PCR sequencing |
| **P_219ID_** | F: TGAAACTTTTGGCAAGCATGA  R: CTGTGTCTCCTCGTGAGTCC | chr1: 1,976,113 – 1,976,133  chr1: 1,976,318 - 1,976,299 | Ref. [18] | 59 °C | Agarose gel electrophoresis |

**Note**: The binding locations of forward (F) and reverse (R) primers were relatively scored to UMD3.1 release of the *B. taurus* genome or mitochondrial genome (GenBank accession no. V00654). The capillary electrophoresis was conducted an ABI PRISM 3730 XL DNA Analyzer (Applied Biosystems).
